# Towards Detecting Pneumonia Progression in COVID-19 Patients by Monitoring Sleep Disturbance Using Data Streams of Non-Invasive Sensor Networks

**DOI:** 10.3390/s21093030

**Published:** 2021-04-26

**Authors:** Ace Dimitrievski, Eftim Zdravevski, Petre Lameski, María Vanessa Villasana, Ivan Miguel Pires, Nuno M. Garcia, Francisco Flórez-Revuelta, Vladimir Trajkovik

**Affiliations:** 1 Faculty of Computer Science and Engineering, Ss. Cyril and Methodius University, 1000 Skopje, Macedonia; eftim.zdravevski@finki.ukim.mk (E.Z.); petre.lameski@finki.ukim.mk (P.L.); trvlado@finki.ukim.mk (V.T.); 2 Faculty of Health Sciences, Universidade da Beira Interior, 6200-506 Covilhã, Portugal; maria.vanessa.villasana.abreu@ubi.pt; 3 Instituto de Telecomunicações, Universidade da Beira Interior, 6200-001 Covilhã, Portugal; impires@it.ubi.pt (I.M.P.); ngarcia@di.ubi.pt (N.M.G.); 4 Computer Science Department, Polytechnic Institute of Viseu, 3504-510 Viseu, Portugal; 5 UICISA:E Research Centre, School of Health, Polytechnic Institute of Viseu, 3504-510 Viseu, Portugal; 6 Department of Computer Technology, Universidad de Alicante, 03690 Alicante, Spain; francisco.florez@gcloud.ua.es

**Keywords:** COVID-19, sensors, connected healthcare

## Abstract

Pneumonia caused by COVID-19 is a severe health risk that sometimes leads to fatal outcomes. Due to constraints in medical care systems, technological solutions should be applied to diagnose, monitor, and alert about the disease’s progress for patients receiving care at home. Some sleep disturbances, such as obstructive sleep apnea syndrome, can increase the risk for COVID-19 patients. This paper proposes an approach to evaluating patients’ sleep quality with the aim of detecting sleep disturbances caused by pneumonia and other COVID-19-related pathologies. We describe a non-invasive sensor network that is used for sleep monitoring and evaluate the feasibility of an approach for training a machine learning model to detect possible COVID-19-related sleep disturbances. We also discuss a cloud-based approach for the implementation of the proposed system for processing the data streams. Based on the preliminary results, we conclude that sleep disturbances are detectable with affordable and non-invasive sensors.

## 1. Introduction

Coronavirus disease (COVID-19) is an acute infectious disease caused by Severe Acute Respiratory Syndrome (SARS-CoV) [1]. The authors of [2] reported the discovery of SARS-CoV-2 in December 2019 in Wuhan, China. It is a sometimes deadly disease that mostly affects elderly patients and patients with specific comorbidities, the most frequent of which include hypertension, diabetes, severe asthma, respiratory disease, and cardiovascular disease [3,4].

Tang et al. [5], reported that hospitalized patients mostly have cases of pneumonia, which is the leading cause of fatal failures in the respiratory and cardiac systems [6]. Clinical observations show that the COVID-19 disease can rapidly progress, with a period from hospitalization to death of 15.9 days (standard deviation = 8.8 d) and 12.5 days (8.6 d, *p* = 0.044) for intensive care unit (ICU) patients and non-ICU patients, respectively [6]. The disease can rapidly worsen, leading to respiratory failure and acute respiratory distress syndrome (ARDS), which requires intubation [7].

Due to the medical systems’ capacity constraints in areas where the disease is widely spread, supportive care and patient monitoring are limited. Early detection of pneumonia development in patients in self-isolation at home could enable medical staff evaluation and timely admission to hospital care.

Patients with medium and severe disease experience deterioration in their wellbeing. Symptoms include cough, fever, dyspnea, musculoskeletal symptoms (joint pain, fatigue), and gastrointestinal symptoms [8]. Based on our earlier research [9,10,11], we propose a method for non-invasive monitoring of sleep disturbances, as developing pneumonia could affect a person’s breathing and quality of sleep. To establish our assumption that at-home patient monitoring, specifically sleep monitoring, could detect the worsening of the situation of COVID-19 patients or establish if they present a higher risk, in this paper, we review the literature on the relations between COVID-19 and sleep, as well as technology-aided patient monitoring.

In the next section, we provide a review of the literature on the relation between COVID-19, as well as its effect on sleep, and technology-aided patient monitoring. In Section 3, we describe our scenario for non-invasive sleep monitoring, propose a cloud-based approach for sleep disturbance detection, and outline the process for building machine learning (ML) models for detecting sleep disturbances that might indicate underlying COVID-19 issues. The results from the experiments are presented in Section 4. We discuss our findings and future work in Section 5, and conclude the paper in Section 6.

## 2. Related Work

To establish our assumption that at-home patient monitoring, specifically sleep monitoring, could detect the worsening of the situation of COVID-19 patients or establish if they present a higher risk, in this section, we review the literature on the relations between COVID-19 and sleep, as well as technology-aided patient monitoring.

### 2.1. COVID-19 and Sleep Disturbances

COVID-19-associated ARDS imposes hypoxia [12], which is an indication of the development of more progressive pneumonia. Patients with hypoxia require urgent medical attention. Smartphone pulse oximetry has been used to detect hypoxia. While pulse oximetry is a direct way to detect hypoxia [13], it has the limitation that the patient must adequately use the equipment and know how to take measurements. It is also challenging to ensure that a person can keep the pulse oximeter attached to their finger during sleep. Due to the lack of oxygen saturation, hypoxia causes sleep disturbances [14]. Sleep monitoring can thus detect potential hypoxia. While false positives from other causes affecting sleep are possible, a further pulse oximetry measurement by the patient or another caregiver can be used for confirmation.

Another aspect of how sleep monitoring could benefit from accessing risk factors for COVID-19 patients is by observing the effects of comorbidities. McEvoy [15] showed that overnight oxygen deprivation caused by obstructive sleep apnea syndrome is a strong predictor of hypertension. Therefore, by extension, obstructive sleep apnea syndrome (OSA) is an indicator of at least one risk factor for COVID-19 patients.

Yi-Fong Su et al. [16] observed 34,100 patients, of which 2757 patients had pneumonia during a mean follow-up period of 4.5 years. This study showed that patients with obstructive sleep apnea syndrome experience a 1.20-fold increase in incident pneumonia. Thus, obstructive sleep apnea syndrome appears to confer a higher risk for future pneumonia. We did not find any similar studies that were specifically for COVID-19 patients; however, Pazarli et al. [17] postulated that OSA may be a risk factor for mortality or deteriorate the clinical scenario with COVID-19. McSharry et al. [18] suspected that OSA could potentially contribute to worsening hypoxemia and the cytokine storm that occurs in COVID-19 patients. Our approach for detecting obstructive sleep apnea syndrome symptoms could benefit in the diagnosis of this risk factor.

Patients with pneumonia who are not on mechanical ventilation are usually positioned so that the affected areas of the lungs are on top [19]. In [9], we showed that non-invasive sensors could be used to recognize motions in bed, including turning in bed from lying on the back to laying on the side. Detecting such movements could alert the caregiver to monitor the care receiver and, if needed, change their body position.

### 2.2. Technology-Aided Patient Monitoring

Improvements in healthcare combined with an aging population with a greater need for health services have strained hospitals and medical staff, which are not always at scale with the needed capacity. This effect has been partially reduced by reducing the length of stay in inpatient hospitals for some patients [20,21]. On the other hand, the tendency to reduce the length of stay in hospitals, which also reduces exposure to hospital-acquired diseases, has created a need for at-home patient monitoring and care. Active monitoring of patients in home settings can improve adherence for patients receiving care at home [22].

Patient monitoring is a growing field of research, and various designs and systems have been proposed. A comprehensive review of remote patient monitoring was conducted in [23]. This study focused on four categories, one of which was cardiovascular and respiratory-related diseases. The review shows that this technology is making an impact on society and the research community. The authors noted that, although researchers prefer to move towards contactless methods, there are still significant problems to be solved in contactless monitoring. These problems include adapting systems for different users and removing artifacts and noise from the contactless sensors. Vegesna et al. [24] conducted a systematic review of remote patient monitoring using non-invasive technologies. This study showed that most systems use multiple components, and smartphones are often involved.

A collaborative healthcare system (COHESY) model was described in Kotevska et al. [25]. This model had a bio-network layer for collecting sensor data, a social layer, and a layer for interoperability with healthcare information systems. This system addressed data security issues, such as authentication, privacy, data storage, transmission, and confidentiality.

A system for unobtrusive monitoring of sleep and respiration was proposed in Choi et al. [26]. According to the researchers, the system that used a thin strip pressure sensor to measure the care receiver’s sleep efficiency and respiration rate had an accuracy similar to that of existing Food and Drug Administration (FDA)-approved sleep trackers. Two sensors were used in this study: The first one used the piezoelectric effect, and the second was a force-sensing resistor. Once the analog signals were converted into digital signals, they were sent via Bluetooth to a smartphone and onward to an internet server.

Another approach for obstructive sleep apnea syndrome monitoring and detection is through nocturnal pulse oximetry. This approach, studied in Hwang et al. [27], where the authors showed an accuracy of diagnosis of 96.7%. Though the study was done in a hospital setting, the paper showed the potential for home-based use of connected pulse oximetry.

While pulse oximetry provides a more accurate diagnosis for obstructive sleep apnea syndrome, there are many challenges with training care receivers to properly put on the device and consistently do that before sleep. Wearable devices can also fall off or cause discomfort to the patient. Given these downsides, the unobtrusive monitoring with devices that require little or no human intervention can be a more consistent way to measure sleep patterns and sleep disturbances. Shao et al. developed a non-contact blood oxygen saturation ($SpO_{2}$) monitoring system using camera and dual-wavelength imaging [28]. Their method uses a complementary metal–oxide–semiconductor (CMOS) camera to record photoplethysmography (PPG) signals alternately at two particular wavelengths corresponding to orange and near infrared. Another system for contactless monitoring of blood oxygen saturation was proposed in Casalino et al. [29]. The authors used the plethysmographic signal obtained by monitoring the areas of interest on a person’s face to derive an estimation of $SpO_{2}$.

## 3. Methods

Our proposed solution consists of non-invasive sensors. We utilize two types of sensors: a piezoelectric sensor (Angelcare, Montreal, QC, Canada) and passive infrared (PIR) sensors (Sunfounder, Shenzhen, China). Other data sources, including patient input and digital medical records, are also introduced into the system. We present a cloud-based architecture to support the care receivers and care providers.

### 3.1. Sensor Kit with Non-Invasive Sensors for Sleep Monitoring

**Figure 1:**
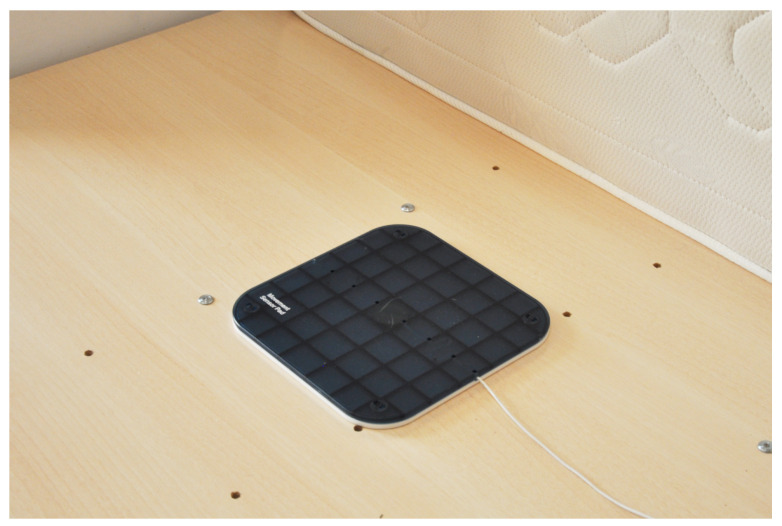
Piezoelectric-based sensor placed under the mattress to detect movements in bed.

Non-invasive sensors can detect body or leg movements. According to Somers et al. [30], these movements are related to obstructive sleep apnea syndrome. Thus, we propose placing piezoelectric sensors under a mattress, as presented in Figure 1. In this figure, a piezoelectric element is placed between two plates. The piezoelectric element generates a charge, which is amplified by a charge amplifier circuit transmitted via a wall connector to the central panel. The plates were used to amplify the movement of a person in a bed. This sensor’s signal was then amplified using a circuit with the schematic shown in Figure S1.

**Figure 2:**
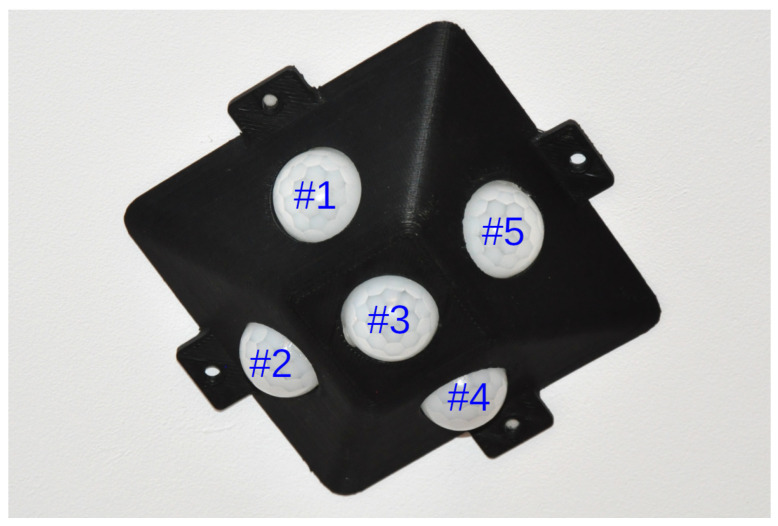
Sensor module with passive infrared (PIR) sensors.

Other sensors were also used, such as a passive infrared (PIR) sensor module [9] placed above the bed, as shown in Figure 2. These sensors were placed in a sensor case to provide the experiment with repeatability with a predetermined angle. They were used to detect events, including movements in the bed that were readable by the under-mattress sensors.

### 3.2. Cloud-Based Architecture

To support this study’s goals, we propose a cloud-based solution that integrates data from various sources. The cloud infrastructure can also facilitate scalability with the resource demand and cost optimization and can simplify deployments to other locations [31,32]. However, the module for data collection and basic processing should be implemented on an edge device [10] and then sent to the cloud. The sensors can have direct wireless internet connectivity and upload information directly to the cloud in a scenario where cost savings are the priority. However, utilizing the healthcare gateway as an edge device is preferable to offloading initial data processing and enabling faster scaling. Such pre-processing can include basic validation of the user inputs by making sure that they are within reasonable ranges, or more advanced processing based on machine learning, signal processing, or time series analysis algorithms. For example, such methods can be implemented within a mobile application on the edge to identify patterns and measure the results of various functional tests, such as the Heel-Rise test [33], Sit-to-Stand test [34], Functional Reach Test [35], or the Timed Up and Go test [36]. In some cases, these tests’ results could be directly calculated within the mobile app and then provided as already-processed results to the cloud.

**Figure 3:**
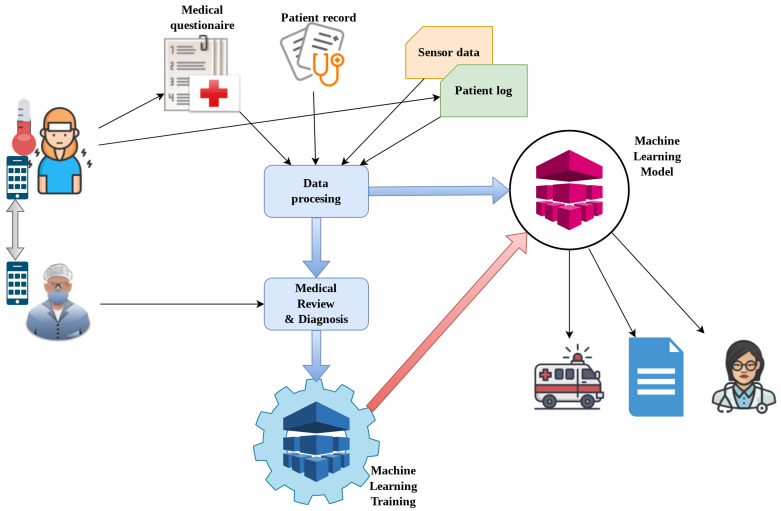
High-level data collection and service delivery flow.

The process for machine learning is presented in Figure 3. The inputs of the system are sensor data, patient logs, patient records, and medical questionnaires. Ideally, most data inputs would be integrated or facilitated through an intuitive user interface within a mobile application. The sensor data collection is elaborated upon in Section 3.1 and can be enhanced with various functional tests, as mentioned in the previous paragraph.

The patient log consists of self-reporting of measurable health parameters, such as body temperature and pulse oximetry. The patient record refers to the medical history of the patient, including any respiratory or sleep diseases. The questionnaire is filled out by the patient. The questions refer to health status that cannot directly be measured, and are thus subjective. Typical questions would include qualifying the person’s sleep and a symptom chart for common COVID-19 symptoms, such as loss of smell and dry cough. A medical professional can then provide their diagnosis or introduce additional parameters, such as relevant parameters from blood tests, electrocardiography (ECG) tests, and electroencephalography (EEG) tests [37]. Physicians could also request an examination by directly making an appointment with the patient or requesting additional manual measurements (e.g., pulse oximetry, blood pressure, or temperature) using connected devices or manual input. In case the patient’s health is worsening or the health risk is increasing, the medical providers can be alerted or suspected events can be added to the medical record.

### 3.3. Machine Learning with Multi-Modal Data

**Figure 4:**
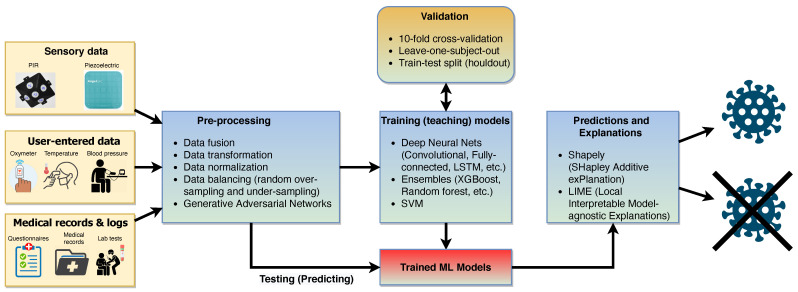
Steps in the machine learning process.

The diverse inputs can potentially lead to a rich feature set that can be used for training machine learning models. Once the data are collected in the cloud, the machine learning process needs to execute several phases to tackle different challenges that are needed to make accurate predictions. Figure 4 presents the flow of machine learning steps needed to detect sleep disturbances using non-invasive sensors (the PIR module and piezoelectric sensor) and other inputs while considering data from multiple care recipients.

As the non-invasive sensors do not make direct measurements, their placement affects how the events are registered. This introduces challenges in generating machine learning models from multiple care receivers. However, when multiple sensors of the same type are used, the data variations and a temporal difference for the same sensor can be introduced as features in the model. The measured features should be invariant to amplitude or time shifting, uniform amplification, additive noise, and time-scaling transformations [38]. This requires considerable pre-processing for data fusion and data type standardization, considering that there are multi-modal data. First, there are sensory data in time-series format, thus requiring feature extraction and alignment, which could be done with time-series analysis approaches, such as that of Zdravevski et al. [39], or by using deep learning approaches, such as that of Ordónez and Roggen [40]. For medical records, questionnaires, and other nominal data, approaches like that of Zdravevski et al. [41], which convert these data into numeric data, are required. These diverse data streams need to be fused, which poses another set of challenges, as eloquently described in [42]. Another challenge is class imbalance, which is common in medical datasets [43]. Traditionally, it has been tackled with the random over-sampling, under-sampling, or SMOTE (Synthetic Minority Oversampling Technique) methods [44]. Recently, generative adversarial networks have been employed for such tasks [45]. Choices are abundant for the machine learning algorithms, and the state-of-the-art and future challenges and research directions for medical datasets were described in Kalantari et al. [46]. Almost all algorithms require some hyper-parameter tuning [47], and their performances can significantly vary on the choice of the cross-validation approach. In the case of medical datasets, which often exhibit a high class imbalance, a leave-one-subject approach is preferable, leading to an overall reliable estimation of the classifier performance [48]. Finally, after the trained machine learning models are deployed on the cloud, they will be employed to make predictions of patients’ health. In medical applications, the explainability of predictions is paramount [49], which is the reason for the trending research topic of explainable artificial intelligence (XAI). Two of the most popular methods for explaining the predictions of traditional black-box models are SHAP (Shapley Additive exPlanation) and LIME (Local Interpretable Model-agnostic Explanations), which also have open-sourced libraries that are integrated into other machine learning toolkits [50].

## 4. Results

The sampling rate was set to 33 Hz, providing readings from five PIR sensors and one piezoelectric sensor every 30 milliseconds. The different experiments for the monitoring of sleep patterns were performed for over 8 h. The PIR sensors were binary, and in the dataset, they could have a value of zero or one. The piezoelectric sensor used an analog input with a voltage from zero to five volts, represented as zero to 1000. For the analysis, we normalized this range between zero and one. The input range was less than the five volts due to signal noise and voltage drop from the amplifier circuit. The summary of the sensor data input is shown in Table 1. Here, we notice that PIR1 and PIR5, which were facing away from the subject, had a low activation rate compared to the other sensors.

**Figure 5:**
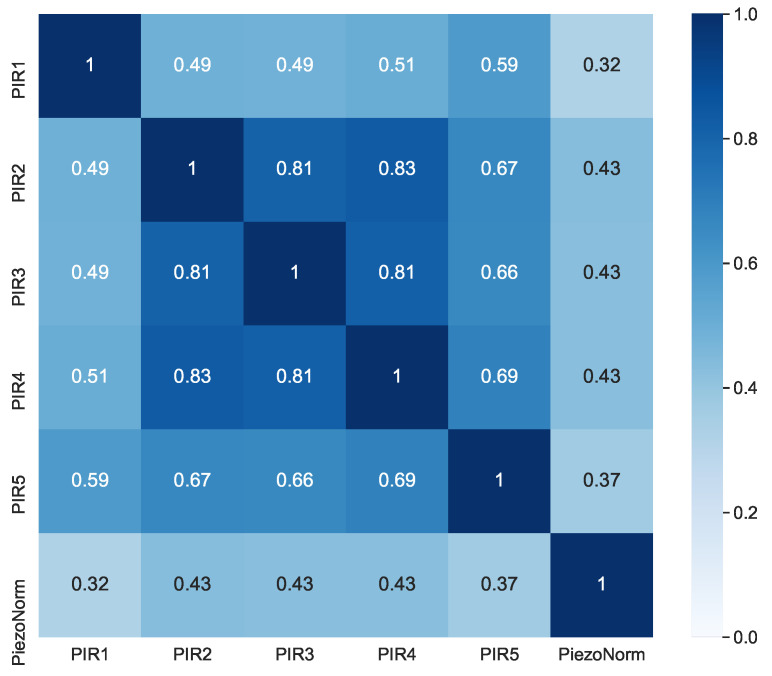
Correlation heatmap of the piezoelectric and passive infrared (PIR) sensors.

Figure 5 shows the correlation between the different PIR sensors and the piezoelectric sensor. It is quite interesting that all of them are significantly correlated.

**Figure 6:**
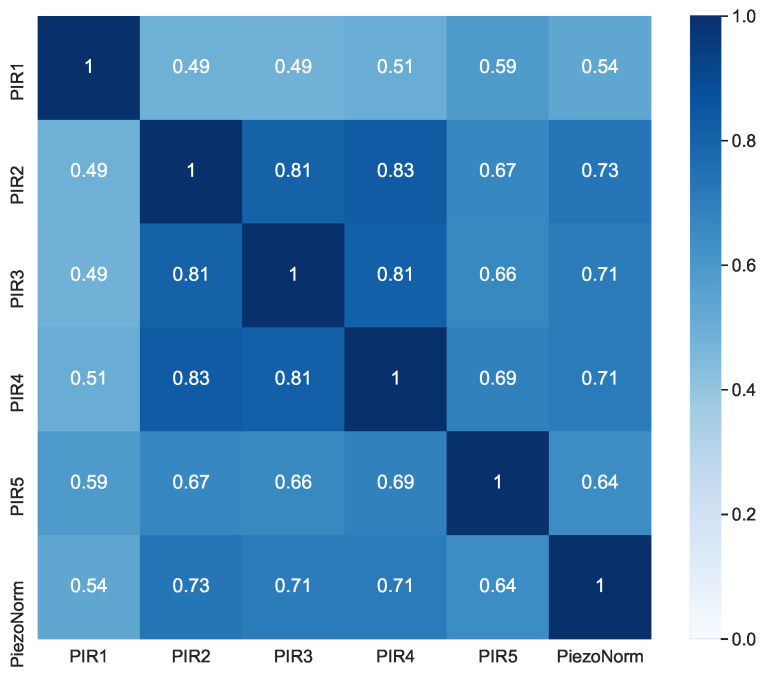
Correlation heatmap of the PIR and piezoelectric sensors with a sliding window.

Suppose that we consider a built-in delay in the PIR sensors and a highly oscillating output of the piezoelectric sensor, thus reducing the correlation. In that case, the calculated correlation is very promising. Post-processing of the data can partially eliminate these factors. The delay of the PIR sensors can be reduced by eliminating successive positive values in the time series. The piezoelectric signal oscillations can be ironed out using the sliding window method and then normalizing each event. Figure 6 shows the heat map, where the piezoelectric data were averaged using a sliding window of 100 samples or 3.3 s.

The results showed a very high correlation among the PIR sensors of up to 0.83. This is understandable considering that the five PIR sensors had different fields of view, and the pairs of sensors that both captured a portion of the bed in its field of view were the ones with higher correlation. Likewise, there was a very high correlation of up to 0.73 between the piezoelectric sensor and the second PIR sensor, which entirely captured the bed in its field of view. This is quite remarkable considering that PIR sensors produced only binary signals, whereas the piezoelectric sensor had almost direct contact with the person. Detecting this temporal correlation of both sensor types implicates that they can be combined to reduce false positives and increase the confidence level for detecting movements in the bed.

**Figure 7:**
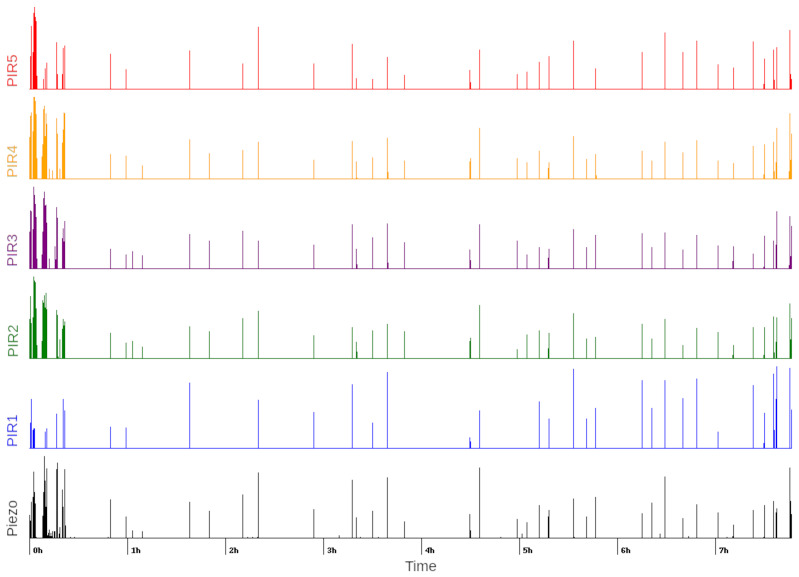
PIR and piezoelectric sensor activation (over 8 h of sleep).

To explore the data in greater detail, in Figure 7, we have visualized the entire sleep interval. As we had close to a million data points for each sensor, we averaged each 30 s interval. Since the sampled data mostly had values of zero, we normalized the data. Each line represents 30 s in the figure, and the vertical length of the line represents the normalized average for that interval. It can be observed that most events detected by the piezoelectric sensor were detected by at least a few of the PIR sensors.

**Figure 8:**
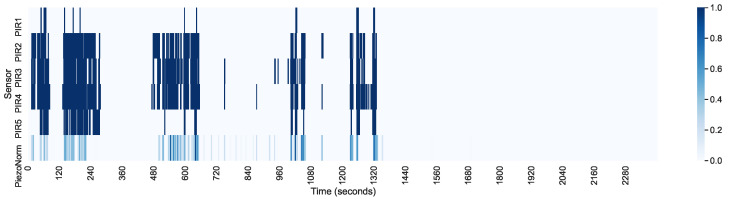
PIR and piezoelectric sensor activation (the first 40 min of the 8 h of sleep).

**Figure 9:**
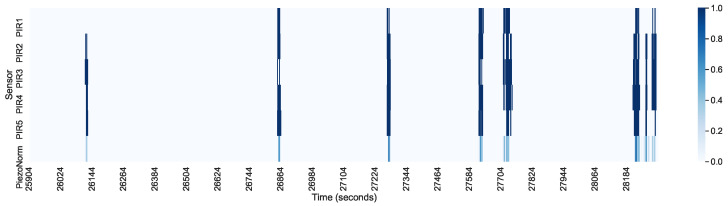
PIR and piezoelectric sensor activation (the last 40 min of the 8 h of sleep).

In Figure 8 and Figure 9, we present a heatmap of the sensors for the first and the last 40 min of the sleep interval. A rolling window was used to average the signal, especially from the piezoelectric sensor. We notice that PIR2, PIR3, and PIR4 were activated even for weaker signals from the piezoelectric sensor. These sensors faced the person at an angle with higher sensitivity. When this signal was stronger, which corresponded with more pronounced body movement, even the PIR1 and PIR5 sensors were activated.

We can conclude that non-invasive sensors are likely to register movements during sleep, as indicated by the high correlation. After labeling data using body sensors, the model would process and react only to non-invasive sensors.

## 5. Discussion

The proposed non-invasive sleep monitoring system cannot directly be used for COVID-19 diagnosis and is not a replacement for professional hospital monitoring for critically ill patients. However, in situations where the patients are at home, our system can be easily placed in a bedroom to monitor if the patient’s situation has an increased probability of worsening by affecting their sleep. Our approach can gather some of the data points needed to further investigate the effects of COVID-19 symptoms and how they affect sleep. However, clinical observation is also needed to precisely monitor the progress of the illness in patients and as a feedback loop to validate the hypothesis that COVID-19 symptoms affect sleep. A machine learning approach is a good fit for this type of analysis given the amount of sensor data generated.

Our system can also be used as an indication of potential risk factors, such as obstructive sleep apnea syndrome. In the related work, we presented research indicating the correlation between sleep disturbances and known effects in patients with COVID-19. A significant association between obstructive sleep apnea syndrome and COVID-19 death was found in Cade et al. [51]. This finding persisted when data were adjusted for demographics. The authors highlighted the need for close monitoring of persons with infection who suffered from obstructive sleep apnea syndrome. The hypoxia associated with OSA significantly affects patients with pneumonia and shortness of breath. The frequent periods of awakening during sleep result in sleep deprivation and poor sleep quality, which are associated with suppression in immune response, which can facilitate susceptibility to SARS-CoV-2 infection [52]. OSA was associated with an increased risk of hospitalization and approximately doubled the risk of developing respiratory failure [53]. Given these risk factors and knowing that OSA is widely under-diagnosed [53], our approach can provide additional information for care providers to investigate and assess a patient’s risk.

In our prior research [9], we demonstrated that the data captured by piezoelectric sensors are correlated with a person’s movements in a bed by using a video camera. Due to the highly intrusive nature of video cameras, they are inappropriate for obtaining ground-truth data about movements in bed during sleep on a larger scale. Therefore, by confirming that piezoelectric sensor data accurately represent movements in bed, data from piezoelectric sensors can be considered as the ground truth. Even though piezoelectric sensors are inexpensive, the PIR sensors are even more affordable and less intrusive, so in this study, we aimed to utilize them to detect movements in bed. Consequently, they could later be used as indicators of other medical conditions. The experiments showed a strong correlation between the PIR sensors and the piezoelectric sensors. These sensors had entirely different measuring methods, confirming the sensor fusion approach’s validity in unobtrusive patient monitoring.

While increased movements during sleep can be associated with COVID-19-related risk factors, such as obstructive sleep apnea and symptoms like hypoxia, restless sleep can be a symptom of other conditions. The stress and fear of being positive for COVID-19 in themselves might affect a person’s sleep. The characteristics of the body movements, including frequency, duration, intensity, and the sleep period in which they occur, can be used as input for machine learning models in order to distinguish types of sleep disturbances and identify whether they originate from an illness. Validating this with actual patients with COVID-19 is out of the scope of this work, as this work focuses on establishing the framework and methodology for such subsequent experiments. Considering all safety regulations and recommendations, performing such experiments during the pandemic is simply unfeasible.

Another application that our non-invasive sleep monitoring approach could benefit is the long-term home-care monitoring of patients who survived the acute respiratory distress syndrome (ARDS) and recovered after mechanical ventilation. Prior research has shown that sleep disturbance can increase in post-recovery ARDS patients compared to the general population [54,55]. Lee et al. [54] followed a large group of patients who survived critical illness associated with ARDS and concluded that chronic sleep disorders that originate during the acute illness are present in some ARDS survivors several months after discharge from the hospital. Based on their study and research in the literature, Doria et al. [55] found that by median percentage, 67% of patients in the early stage and 39% in the late stage after discharge experience abnormal sleep.

An additional benefit of using our approach is the assistance in the monitoring of patients with sleep disorders. Many sleep disorder centers were entirely closed during the COVID-19 pandemic either because they were situated in hospital buildings or because the staff was re-tasked with COVID-19 care [56]. While therapy for obstructive sleep apnea syndrome using positive airway pressure (PAP) devices is usually administered at home, sleep monitoring is done in these centers. Given the increased limitations and restrictions, the role of telemedicine for sleep disorders should be prioritized in the era of COVID-19 [57].

## 6. Conclusions

In this paper, we showed links between COVID-19 symptoms and sleep disturbances. We presented a system consisting of multiple sensors of two types to monitor sleep quality and issues. Our experimental data showed a strong correlation between diverse types of sensors that detect movements during sleep. We discussed the relations found in the literature between movements in sleep, sleep quality, and sleep disturbances. The monitoring of sleep and sleep disturbances, in turn, can indicate the existence of COVID-19 symptoms, including pneumonia and possible COVID-19 risk factors, such as obstructive sleep apnea syndrome. Our approach can also be used as an alternative home-based sleep monitoring when the patient cannot receive specialized monitoring in sleep centers due to the pandemic restrictions. In the future, we will collect data across multiple persons and various placement configurations of non-invasive sensors.

## Figures and Tables

**Table 1:** Summary of the sensor readings.

Sensor	Min	Mean	Max
Piezoelectric	37	52.03	736
Piezoelectric (normalized)	0	0.021503	1
PIR1	0	0.009828	1
PIR2	0	0.028537	1
PIR3	0	0.029203	1
PIR4	0	0.030796	1
PIR5	0	0.018591	1

## Data Availability

The data utilized in this study is freely available at https://github.com/acemk/sleep_monitoring/raw/main/bedlog2.zip.

## Supplementary Materials

The following are available online at https://www.mdpi.com/article/10.3390/s21093030/s1, Figure S1: Amplifier circuit for the piezoelectric sensor.

## Abbreviations

The following abbreviations are used in this manuscript:

COVID-19	Coronavirus disease 2019
SARS-CoV-2	Severe acute respiratory syndrome—coronavirus 2
ARDS	Acute respiratory distress syndrome

## References

[1] Pires I.M. A review on Diagnosis and Treatment methods for coronavirus disease with sensors. Proceedings of the 2020 International Conference on Decision Aid Sciences and Application (DASA).

[2] Tang X., Wu C., Li X., Song Y., Yao X., Wu X., Duan Y., Zhang H., Wang Y., Qian Z. (2020). On the origin and continuing evolution of SARS-CoV-2. Natl. Sci. Rev..

[3] Grasselli G., Zangrillo A., Zanella A., Antonelli M., Cabrini L., Castelli A., Cereda D., Coluccello A., Foti G., Fumagalli R. (2020). Baseline characteristics and outcomes of 1591 patients infected with SARS-CoV-2 admitted to ICUs of the Lombardy Region, Italy. JAMA.

[4] Williamson E.J., Walker A.J., Bhaskaran K., Bacon S., Bates C., Morton C.E., Curtis H.J., Mehrkar A., Evans D., Inglesby P. (2020). Factors associated with COVID-19-related death using OpenSAFELY. Nature.

[5] Weiss P., Murdoch D.R. (2020). Clinical course and mortality risk of severe COVID-19. Lancet.

[6] Du R.H., Liu L.M., Yin W., Wang W., Guan L.L., Yuan M.L., Li Y.L., Hu Y., Li X.Y., Sun B. (2020). Hospitalization and critical care of 109 decedents with COVID-19 pneumonia in Wuhan, China. Ann. Am. Thorac. Soc..

[7] Goh K.J., Kalimuddin S., Chan K.S. (2020). Rapid progression to acute respiratory distress syndrome: Review of current understanding of critical illness from coronavirus disease 2019 (COVID-19) infection. Ann. Acad. Med. Singapore.

[8] Carfì A., Bernabei R., Landi F. (2020). Persistent symptoms in patients after acute COVID-19. JAMA.

[9] Dimitrievski A., Zdravevski E., Lameski P., Trajkovik V. Towards application of non-invasive environmental sensors for risks and activity detection. Proceedings of the 2016 IEEE 12th International Conference on Intelligent Computer Communication and Processing (ICCP).

[10] Dimitrievski A., Zdravevski E., Lameski P., Goleva R., Koceski S., Trajkovik V. Fog Computing for Personal Health Principles. Proceedings of the 8th International Conference on Applied Internet and Information Technologies.

[11] Dimitrievski A., Savoska S., Trajkovikj V. Fog Computing for Personal Health: Case Study for Sleep Apnea Detection. Proceedings of the 13th Conference on Information Systems and Grid Technologie.

[12] Kashani K.B. (2020). Hypoxia in COVID-19: Sign of Severity or Cause for Poor Outcomes. Mayo Clinic Proceedings.

[13] Teo J. (2020). Early Detection of Silent Hypoxia in Covid-19 Pneumonia Using Smartphone Pulse Oximetry. J. Med Syst..

[14] Mizuno K., Asano K., Okudaira N. (1993). Sleep and respiration under acute hypobaric hypoxia. Jpn. J. Physiol..

[15] McEvoy R.D. (2014). Obstructive Sleep Apnoea and Hypertension: The ESADA Study. Eur. Respir. J..

[16] Su V.Y.F., Liu C.J., Wang H.K., Wu L.A., Chang S.C., Perng D.W., Su W.J., Chen Y.M., Lin E.Y.H., Chen T.J. (2014). Sleep apnea and risk of pneumonia: A nationwide population-based study. Cmaj.

[17] Pazarlı A.C., Ekiz T., İlik F. (2020). Coronavirus disease 2019 and obstructive sleep apnea syndrome. Sleep Breath. Schlaf Atm..

[18] McSharry D., Malhotra A. (2020). Potential influences of obstructive sleep apnea and obesity on COVID-19 severity. J. Clin. Sleep Med..

[19] Froelich S., Mandonnet E., Julla J.B., Touchard C., Laloi-Michelin M., Kevorkian J.P., Gautier J.F. (2020). Towards individualized and optimalized positioning of non-ventilated COVID-19 patients: Putting the affected parts of the lung (s) on top?. Diabetes Metab..

[20] Battleman D.S., Callahan M., Thaler H.T. (2002). Rapid antibiotic delivery and appropriate antibiotic selection reduce length of hospital stay of patients with community-acquired pneumonia: Link between quality of care and resource utilization. Arch. Intern. Med..

[21] Clarke A. (2002). Length of in-hospital stay and its relationship to quality of care. BMJ Qual. Saf..

[22] Fox N., Hirsch-Allen A., Goodfellow E., Wenner J., Fleetham J., Ryan C.F., Kwiatkowska M., Ayas N.T. (2012). The impact of a telemedicine monitoring system on positive airway pressure adherence in patients with obstructive sleep apnea: A randomized controlled trial. Sleep.

[23] Malasinghe L.P., Ramzan N., Dahal K. (2019). Remote patient monitoring: A comprehensive study. J. Ambient. Intell. Humaniz. Comput..

[24] Vegesna A., Tran M., Angelaccio M., Arcona S. (2017). Remote patient monitoring via non-invasive digital technologies: A systematic review. Telemed. e-Health.

[25] Kotevska O., Vlahu-Gjorgievska E., Trajkovic V., Koceski S. (2012). Towards a patient-centered collaborative health care system model. Int. J. Comput. Theory Eng..

[26] Choi A., Noh S., Shin H. (2020). Internet-Based Unobtrusive Tele-Monitoring System for Sleep and Respiration. IEEE Access.

[27] Hwang S.H., Cho J.G., Choi B.H., Baek H.J., Lee Y.J., Jeong D.U., Park K.S. (2017). Real-time automatic apneic event detection using nocturnal pulse oximetry. IEEE Trans. Biomed. Eng..

[28] Shao D., Liu C., Tsow F., Yang Y., Du Z., Iriya R., Yu H., Tao N. (2015). Noncontact monitoring of blood oxygen saturation using camera and dual-wavelength imaging system. IEEE Trans. Biomed. Eng..

[29] Casalino G., Castellano G., Zaza G. A mHealth solution for contact-less self-monitoring of blood oxygen saturation. Proceedings of the 2020 IEEE Symposium on Computers and Communications (ISCC).

[30] Somers V.K., Dyken M.E., Clary M.P., Abboud F.M. (1995). Sympathetic neural mechanisms in obstructive sleep apnea. J. Clin. Investig..

[31] Zdravevski E., Lameski P., Dimitrievski A., Grzegorowski M., Apanowicz C. Cluster-size optimization within a cloud-based ETL framework for Big Data. Proceedings of the 2019 IEEE International Conference on Big Data (Big Data).

[32] Grzegorowski M., Zdravevski E., Janusz A., Lameski P., Apanowicz C., Slezak D. (2021). Cost Optimization for Big Data Workloads Based on Dynamic Scheduling and Cluster-Size Tuning. Big Data Res..

[33] Pires I.M., Ponciano V., Garcia N.M., Zdravevski E. (2020). Analysis of the Results of Heel-Rise Test with Sensors: A Systematic Review. Electronics.

[34] Marques D.L., Neiva H.P., Pires I.M., Zdravevski E., Mihajlov M., Garcia N.M., Ruiz-Cárdenas J.D., Marinho D.A., Marques M.C. (2021). An Experimental Study on the Validity and Reliability of a Smartphone Application to Acquire Temporal Variables during the Single Sit-to-Stand Test with Older Adults. Sensors.

[35] Pires I.M., Garcia N.M., Zdravevski E. (2020). Measurement of Results of Functional Reach Test with Sensors: A Systematic Review. Electronics.

[36] Ponciano V., Pires I.M., Ribeiro F.R., Marques G., Garcia N.M., Pombo N., Spinsante S., Zdravevski E. (2020). Is The Timed-Up and Go Test Feasible in Mobile Devices? A Systematic Review. Electronics.

[37] Ponciano V., Pires I.M., Ribeiro F.R., Villasana M.V., Canavarro Teixeira M., Zdravevski E. (2020). Experimental Study for Determining the Parameters Required for Detecting ECG and EEG Related Diseases during the Timed-Up and Go Test. Computers.

[38] Esling P., Agon C. (2012). Time-series Data Mining. ACM Comput. Surv..

[39] Zdravevski E., Lameski P., Trajkovik V., Kulakov A., Chorbev I., Goleva R., Pombo N., Garcia N. (2017). Improving Activity Recognition Accuracy in Ambient-Assisted Living Systems by Automated Feature Engineering. IEEE Access.

[40] Ordóñez F.J., Roggen D. (2016). Deep convolutional and lstm recurrent neural networks for multimodal wearable activity recognition. Sensors.

[41] Zdravevski E., Lameski P., Kulakov A., Kalajdziski S. Transformation of nominal features into numeric in supervised multi-class problems based on the weight of evidence parameter. Proceedings of the 2015 Federated Conference on Computer Science and Information Systems (FedCSIS).

[42] Lahat D., Adali T., Jutten C. (2015). Multimodal data fusion: An overview of methods, challenges, and prospects. Proc. IEEE.

[43] Zhang H., Zhang H., Pirbhulal S., Wu W., Albuquerque V.H.C.D. (2020). Active balancing mechanism for imbalanced medical data in deep learning–based classification models. ACM Trans. Multimed. Comput. Commun. Appl. TOMM.

[44] Fernández A., Garcia S., Herrera F., Chawla N.V. (2018). SMOTE for learning from imbalanced data: Progress and challenges, marking the 15-year anniversary. J. Artif. Intell. Res..

[45] Salehinejad H., Valaee S., Dowdell T., Colak E., Barfett J. Generalization of deep neural networks for chest pathology classification in X-rays using generative adversarial networks. Proceedings of the 2018 IEEE International Conference on Acoustics, Speech and Signal Processing (ICASSP).

[46] Kalantari A., Kamsin A., Shamshirband S., Gani A., Alinejad-Rokny H., Chronopoulos A.T. (2018). Computational intelligence approaches for classification of medical data: State-of-the-art, future challenges and research directions. Neurocomputing.

[47] Petrovska B., Atanasova-Pacemska T., Corizzo R., Mignone P., Lameski P., Zdravevski E. (2020). Aerial Scene Classification through Fine-Tuning with Adaptive Learning Rates and Label Smoothing. Appl. Sci..

[48] Li J.P., Haq A.U., Din S.U., Khan J., Khan A., Saboor A. (2020). Heart Disease Identification Method Using Machine Learning Classification in E-Healthcare. IEEE Access.

[49] Tjoa E., Guan C. (2020). A survey on explainable artificial intelligence (xai): Toward medical xai. IEEE Trans. Neural Networks Learn. Syst..

[50] Ramon Y., Martens D., Provost F., Evgeniou T. (2020). A comparison of instance-level counterfactual explanation algorithms for behavioral and textual data: SEDC, LIME-C and SHAP-C. Adv. Data Anal. Classif..

[51] Cade B.E., Dashti H.S., Hassan S.M., Redline S., Karlson E.W. (2020). Sleep apnea and COVID-19 mortality and hospitalization. Am. J. Respir. Crit. Care Med..

[52] Tufik S. (2020). Obstructive Sleep Apnea as a comorbidity to Covid-19. Sleep Sci..

[53] Maas M.B., Kim M., Malkani R.G., Abbott S.M., Zee P.C. (2020). Obstructive Sleep Apnea and Risk of COVID-19 Infection, Hospitalization and Respiratory Failure. Sleep Breath..

[54] Lee C.M., Herridge M.S., Gabor J.Y., Tansey C.M., Matte A., Hanly P.J. (2009). Chronic sleep disorders in survivors of the acute respiratory distress syndrome. Intensive Care Med..

[55] Dhooria S., Sehgal I.S., Agrawal A.K., Agarwal R., Aggarwal A.N., Behera D. (2016). Sleep after critical illness: Study of survivors of acute respiratory distress syndrome and systematic review of literature. Indian J. Crit. Care Med. Peer Rev. Off. Publ. Indian Soc. Crit. Care Med..

[56] Thorpy M., Figuera-Losada M., Ahmed I., Monderer R., Petrisko M., Martin C., Akhtar J., Thorpy J., Haines C. (2020). Management of sleep apnea in New York City during the COVID-19 pandemic. Sleep Med..

[57] Voulgaris A., Ferini-Strambi L., Steiropoulos P. (2020). Sleep medicine and COVID-19. Has a new era begun?. Sleep Med..

