# Supplementary figures and images for: Towards Detecting Pneumonia Progression in COVID-19 Patients by Monitoring Sleep Disturbance Using Data Streams of Non-Invasive Sensor Networks
Source: Sensors (Basel). 2021 Apr 26;21(9):3030. doi: 10.3390/s21093030 (PMC8123511; doi:10.3390/s21093030)

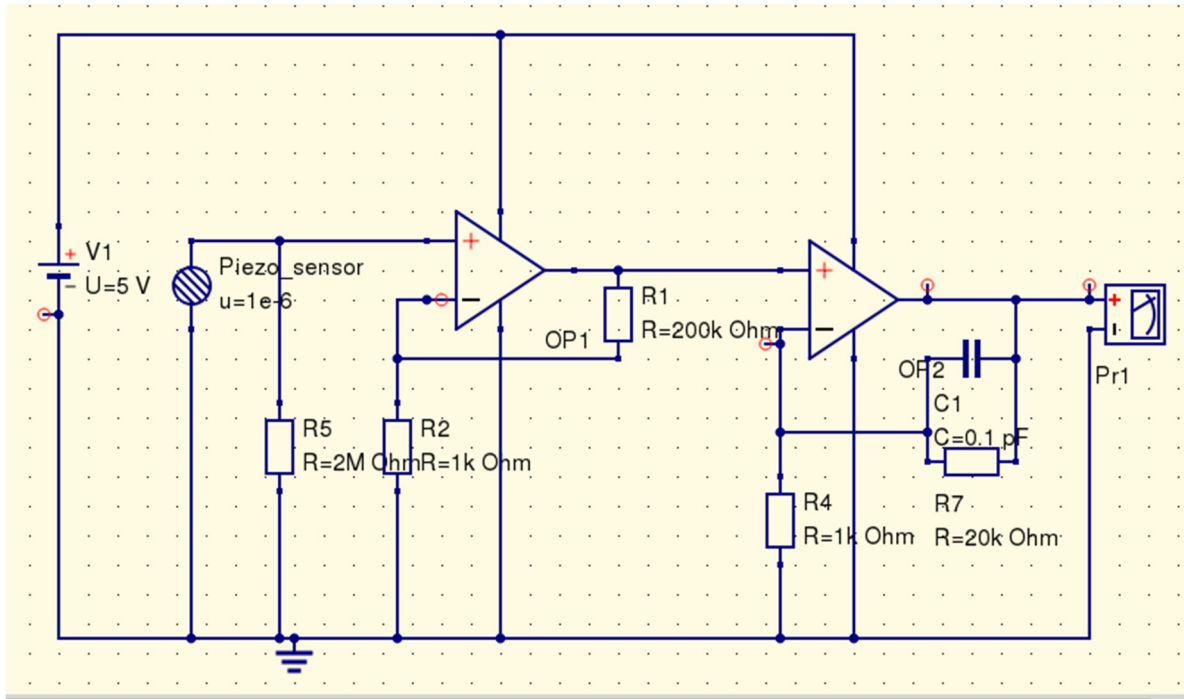

**Figure S1.** Amplifier circuit for piezoelectric sensor.

Supplement: Supplementary file 1 [file sensors-21-03030-s001.zip › sensors-1126811-supplementary.pdf]
